# *Plasmodium knowlesi* and other malaria parasites in long-tailed macaques from the Philippines

**DOI:** 10.1186/s12936-019-2780-4

**Published:** 2019-04-24

**Authors:** Lief Erikson Gamalo, Judeline Dimalibot, Khamisah Abdul Kadir, Balbir Singh, Vachel Gay Paller

**Affiliations:** 1 Animal Biology Division, Institute of Biological Sciences, University of the Philippines Los, Baños, 4031 Los Baños, Laguna Philippines; 2 Present Address: Department of Biological Sciences and Environmental Studies, University of the Philippines Mindanao, Tugbok District, Mintal, 8000 Davao City, Philippines; 3 Malaria Research Centre, Faculty of Medicine and Health Sciences, Universiti Malaysia Sarawak, 94300 Kota Samarahan, Sarawak Malaysia

**Keywords:** Simian *Plasmodium*, *Macaca fascicularis*, Palawan, *Plasmodium knowlesi*

## Abstract

**Background:**

*Macaca fascicularis* (long-tailed macaque) is the most widespread species of macaque in Southeast Asia and the only species of monkey found naturally in the Philippines. The species is the natural host for the zoonotic malaria species, *Plasmodium knowlesi* and *Plasmodium cynomolgi* and for the potentially zoonotic species, *Plasmodium inui*. Moreover, other *Plasmodium* species such as *Plasmodium coatneyi* and *Plasmodium fieldi* are also natural parasites of *M. fascicularis*. The aims of this study were to identify and determine the prevalence of *Plasmodium* species infecting wild and captive long-tailed macaques from the Philippines.

**Methods:**

A total of 95 blood samples from long-tailed macaques in the Philippines were collected from three locations; 30 were from captive macaques at the National Wildlife Rescue and Rehabilitation Center (NWRRC) in Luzon, 25 were from captive macaques at the Palawan Wildlife Rescue and Conservation Center (PWRCC) in Palawan and 40 were from wild macaques from Puerto Princesa Subterranean River National Park (PPSRNP) in Palawan. The *Plasmodium* spp. infecting the macaques were identified using nested PCR assays on DNA extracted from these blood samples.

**Results:**

All 40 of the wild macaques from PPSRNP in Palawan and 5 of 25 captive macaques from PWRCC in Palawan were *Plasmodium*-positive; while none of the 30 captive macaques from the NWRRC in Luzon had any malaria parasites. Overall, *P. inui* was the most prevalent malaria parasite (44.2%), followed by *P. fieldi* (41.1%), *P. cynomolgi* (23.2%), *P. coatneyi* (21.1%), and *P. knowlesi* (19%). Mixed species infections were also observed in 39 of the 45 *Plasmodium*-positive macaques. There was a significant difference in the prevalence of *P. knowlesi* among the troops of wild macaques from PPSRNP.

**Conclusion:**

Wild long-tailed macaques from the island of Palawan, the Philippines are infected with *P. knowlesi, P. inui, P. coatneyi, P. fieldi* and *P. cynomolgi*. The prevalence of these *Plasmodium* spp. varied among the sites of collection and among troops of wild macaques at one site. The presence of these simian *Plasmodium* parasites, especially *P. knowlesi* and *P. cynomolgi* in the long-tailed macaques in Palawan presents risks for zoonotic transmission in the area.

## Background

Over 150 species of *Plasmodium* have been described infecting vertebrate terrestrial animals [1]. Twenty-six species of the genus infect non-human primate hosts [2] and 13 of these infect non-human primates in South East Asia [3]. Four species of *Plasmodium* (*Plasmodium falciparum, Plasmodium vivax, Plasmodium malariae*, and *Plasmodium ovale* spp.), were thought to cause malaria in humans until *Plasmodium knowlesi*, a parasite that infects *Macaca* spp. in nature, was discovered to be commonly infecting humans in the Kapit Division of Sarawak, Malaysian Borneo [4]. Subsequent studies have shown that human cases occur throughout South East Asia [5, 6] and on the Nicobar and Andaman islands of India [7].

Other than *P. knowlesi*, natural human infections with the simian malaria parasite *Plasmodium cynomolgi* have been described in Peninsular Malaysia [8] and Cambodia [9]. A third malaria parasite of macaques, *Plasmodium inui,* was accidentally transmitted to humans in laboratories by mosquito bites and subsequently shown to be infectious under experimental conditions [2]. Thus, monitoring the natural hosts of these non-human primate *Plasmodium* parasites should be given attention as they are the potential reservoir hosts for malaria infections in humans [10–14].

Long-tailed macaques (*Macaca fascicularis*), the only monkey species in the Philippines, are widely distributed in the country, with sightings on the islands of Balabac, Basilan, Biliran, Bohol, Busuanga, Camiguin, Catanduanes, Culion, Leyte, Luzon, northeastern Mindanao, Mindoro, Negros, Panay, Palawan, Samar and Sibuyan [15]. This species can be naturally infected by 6 *Plasmodium* species, namely *P. knowlesi*, *Plasmodium fieldi*, *Plasmodium coatneyi*, *P. cynomolgi*, *P. inui* and *Plasmodium simiovale* [16]. In a recent study, using molecular techniques, *P. cynomolgi* and *P. coatneyi* were detected in long-tailed macaques from Batangas, and *P. cynomolgi, P. coatneyi* and *P. inui* in macaques from Zamboanga, the Philippines [17]. Of all the Philippine islands where *M. fascicularis* are distributed, *P. knowlesi* infected macaques have only been reported previously in Palawan Island in 1978 and Cebu Island in 1961 [18, 19]. Other simian *Plasmodium* species such as *P. inui, P. cynomolgi* and *P. coatneyi*, were described in long-tailed macaques in Palawan island but the observation was solely based on morphological examination of the blood smears from the macaques [19]. With the use of molecular detection methods, human cases of *P. knowlesi* infection have been reported in Palawan Island from five local inhabitants [20] and visitors from Taiwan, Japan and USA [21–23].

The overall aim of this study was to identify *Plasmodium* species and their prevalence in long-tailed macaques (*M. fascicularis*) from Luzon and Palawan Islands. Specifically, the study aimed to identify the *Plasmodium* species in long-tailed macaques using molecular techniques, determine the prevalence of *Plasmodium* parasites from both wild and captive macaques, and compare prevalence of *Plasmodium* spp. among various sites of collection and between different troops of wild macaques.

## Methods

### Collection of samples

**Fig. 1:**
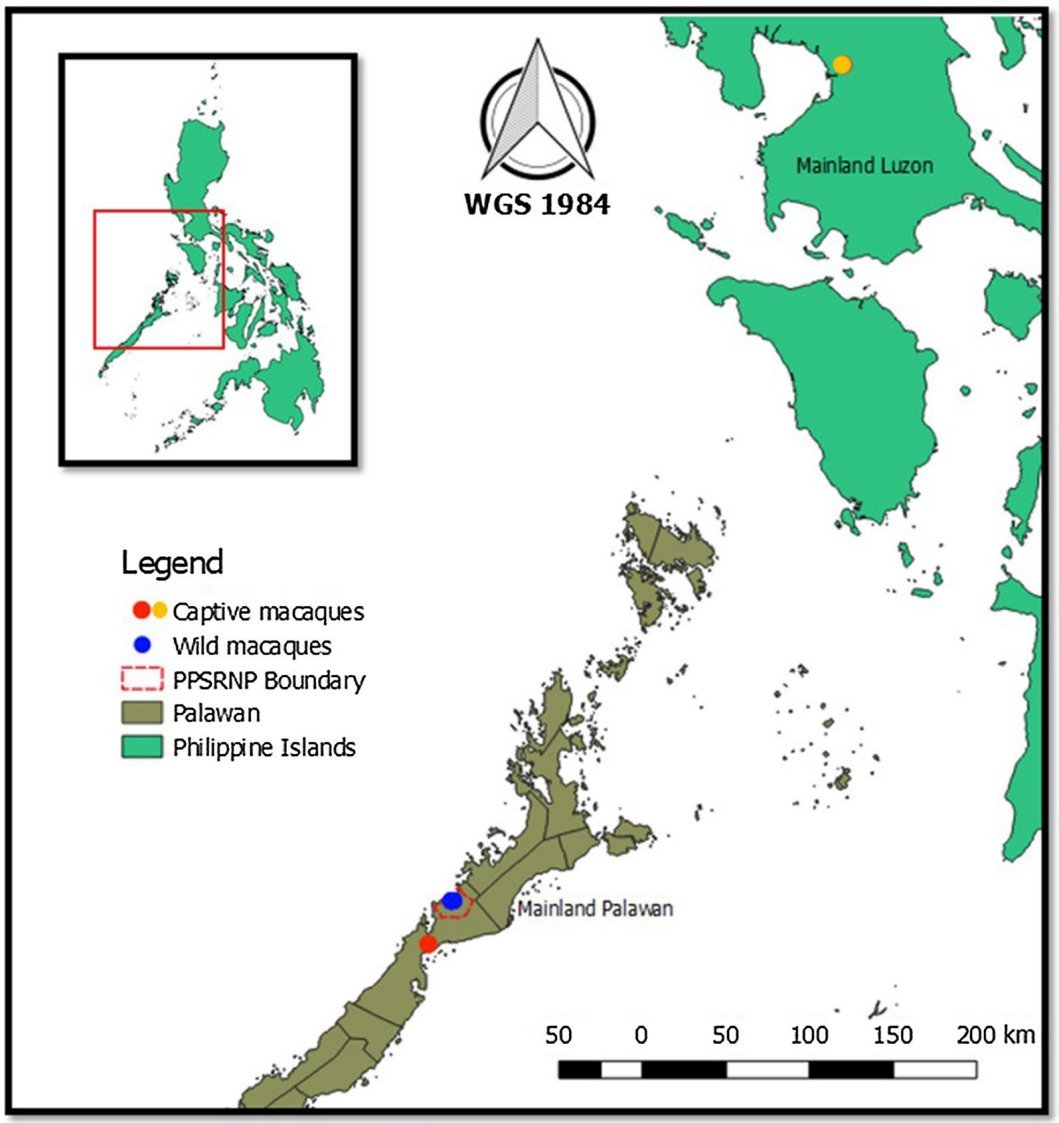
Location of the sampling sites. Wild macaques from Puerto Princesa Subterranean River National Park (PPSRNP) (blue dot), and captive macaques from Palawan Wildlife Rescue and Conservation Center in Palawan (PWRCC) (red dot) and National Wildlife Rescue and Research Center (NWRRC) (orange dot), Philippines. Generated using QGIS version 2.3

A total of 95 blood samples were obtained from long-tailed macaques at the Puerto Princesa Subterranean River National Park (PPSRNP) and the Palawan Wildlife Rescue and Conservation Center (PWRCC) in Palawan, and from the National Wildlife Rescue and Research Center (NWRRC) in Quezon City (Fig. 1) during the month of August and September 2017.

There are approximately 500 free-ranging long-tailed macaques at PPSRNP and humans constantly encounter them. The area is characterized by the presence of beach forest, karst forest, secondary forest and mangrove forest. These diverse habitats offer long-tailed macaques the locations to feed, roost and breed. The area is also near to human settlements and recreation areas. The trapping site was also characterized by presence of park personnel who live within the premises of the Central Park Station. A total of 40 wild macaques were trapped at PPSRNP and they were from three troops (11 from troop 1, 18 from troop 2 and 11 from troop 3). Although troops 1 and 2 have overlapping territories, they have different sleeping sites. The territory of troop 3 was approximately 2 km from that of troops 1 and 2. The 25 macaques at PWRCC and 30 at the NWRRC were all captive macaques. In Palawan, rescued macaques are rehabilitated in the PWRCC located in the South of the Puerto Princesa City. Macaques donated and rescued from the main island Luzon are placed in the NWRRC located in Diliman, Quezon City. Both rescue centers provide shelter for animals confiscated or donated from illegal traders and from private owners.

The macaques were tranquilized, anesthetized intramuscularly with Zoletil (5 mg/kg body weight) before the blood samples were collected as specified in the approved IACUC clearance. Blood samples (maximum of 3 ml per animal) were collected using a syringe from the femoral vein of the macaques into a tube with ethylenediaminetetraacetic acid (EDTA). From the EDTA tubes, three blood spots for each sample were transferred (40–50 μL each) to Whatman 3 MM filter papers in situ. The samples were initially kept at room temperature (20–29 **°**C) for 7–45 days and were transported to ABD Parasitology Laboratory in UPLB and kept refrigerated at 4 °C. Blood spots on filter papers were transported to the Malaria Research Centre, Universiti Malaysia Sarawak, Kota Samarahan, Sarawak, Malaysia for DNA extraction and the molecular analysis.

Gratuitous permits were given by the Palawan Council for Sustainable Development (PCSD) (Permit No.: 2017-07) and from the Biodiversity Management Bureau (Permit No.: 266) to collect blood samples from the island of Palawan and from the NWRRC in Luzon, respectively. Ethics clearance was obtained from the Institutional Animal Care and Use Committee (IACUC) of the University of the Philippines Los Baños (Protocol No.: 2017-0044). The clearance to conduct the study in a protected area was given by the Protected Area Management Board of PPSRNP (Resolution No.: 11-2017). Export and import permits were secured from PCSD (Permit No.: 00003A-2017) and BMB (Permit No.: 23568 A-2017), and Sarawak Forestry Department, Sarawak, Malaysia (Permit No.: 17513), respectively.

### Analysis of samples

DNA was extracted from blood spots in filter papers at the Malaria Research Centre, Universiti Malaysia Sarawak with the use of InstaGene (InstaGene Matrix, Bio-Rad Laboratories, USA) as described previously [24]. This was followed by examining the samples using nested PCR assays with the use of genus and species-specific primers based on the small subunit ribosomal RNA gene. The DNA samples was first examined with the aid of genus-specific primers (rPLU1 and rPLU5, and rPLU3 and rPLU4) as described previously [25]. Positive samples were then examined by nested PCR assays using the species-specific primers to detect *P. knowlesi, P. coatneyi*, *P. cynomolgi, P. inui* and *P. fieldi* as described previously [26]. The products of the amplification were analysed by gel electrophoresis in 2.7% agarose gels and were stained by Sybersafe before being observed under UV light.

### Statistical analysis

Fisher–Freeman–Halton exact test was used to compute the exact probabilities of the differences of the prevalence of *Plasmodium* prevalence rate between habitats (site of collection) and among troops of wild macaques. All statistics were tested using SPSS. Statistical significance for all tests was set at P < 0.05.

## Results

Of the 95 long-tailed macaque samples examined by nested PCR assays, 47.4% were positive for *Plasmodium* spp. (Table 1). Five species of Plasmodium were detected; *P. cynomolgi, P. inui, P. coatneyi P. fieldi* and *P. knowlesi* (Table 1). *Plasmodium inui* was the most prevalent (44.2%), followed by *P. fieldi* (41%), *P. cynomolgi* (23.2%), *P. coatneyi* (21%) and *P. knowlesi* (19%). A majority of the macaques (86.6%; 39/45) had multiple infections; 8 double infections (8.4%), 13 triple (13.7%), 10 quadruple (10.5%), and 8 (8.4%) of them were infected by 5 species of *Plasmodium.*

**Table 1:** *Plasmodium* species infecting long-tailed macaques in Palawan, Philippines

Infection	*Plasmodium* spp.	PPSRNP (n = 40)	PWRCC (n = 25)	NWRRC (n = 30)	Total *Plasmodium*-positive	Prevalence (%)
Single	Pin	2	2	–	4	6.3
	Pk	2	–	–	2	
Double	Pin, Pfld	4	3	–	7	8.4
	Pct, Pfld	1	–	–	1	
Triple	Pcy, Pin, Pfld	7	–	–	7	13.7
	Pct, Pin, Pfld	3	–	–	3	
	Pk, Pin, Pfld	3	–	–	3	
Quadruple	Pcy, Pct, Pin, Pfld	5	–	–	5	10.5
	Pk, Pcy, Pin, Pfld	3	–	–	3	
	Pk, Pct, Pin, Pfld	2	–	–	2	
Quintuple	Pk, Pcy, Pct, Pin, Pfld	8	–	–	8	8.4
Total *Plasmodium*-positive (%)		40 (100)	5 (20)	0 (0)	45 (47.4)	

Pin, *P. inui*; Pk, *P. knowlesi*; Pct, *P. coatneyi*; Pcy, *P. cynomolgi*; Pfld, *P. fieldi*; PPSRNP, Puerto Princesa Subterranean River National Park; PWRCC, Palawan Wildlife Rescue and Conservation Center; NWRRC, National Wildlife Rescue and Research Center

The prevalence of each *Plasmodium* spp. among the macaques varied significantly for the sites of collection. All wild macaques from PPSRNP were infected with malaria parasites compared with only 5 of the 25 (20%) captive macaques from PWRCC and none of the captive ones at NWRRC (Fisher–Freeman–Halton exact test 98.013, P = 0.0001, 95% CI 0.000–0.031). When tested if the prevalence of *P. knowlesi* differs from one macaque troop to another at PPSRNP, troop 2 (Fisher–Freeman–Halton exact test 6.779, P = 0.03, 95% CI 0.000–0.157) showed a lower prevalence compared to troops 1 and 3 (Table 2).

**Table 2:** Comparison of *Plasmodium knowlesi* prevalence among troops of wild macaques in Puerto Princesa Subterranean River National Park, Palawan, Philippines

Troops	Estimated no. of members	Collected samples	Sleeping sites	Prevalence of *P. knowlesi* (%)
1	52	11	Inside forest	63.6
2	43	18	Beside beach	22.2
3	46	11	Inside forest	63.6

## Discussion

In the present study, all of the 40 wild macaques sampled from PPSRNP were infected with *Plasmodium* spp. and there was a 20% infection rate among captive macaques at PWRCC. The presence of *Plasmodium* spp. in PWRCC could suggest that competent vectors could be present at the site. However, it is also possible that the macaques were already infected by *Plasmodium* before they were transported to the rehabilitation centre. No malaria parasites were observed in captive macaques from NWRRC, which is located in Manila, a highly urbanized area. Most of the macaques from these rescue centers were reported to be confiscated or donor pets. The absence of any *Plasmodium* species in macaques in NWRRC could be due to lack of competent vectors of malaria in urbanized areas as what was also observed in Singapore where wild macaques from a forested area had malaria parasites while peri-domestic macaques had none [27]. Similarly in a study in Peninsular Malaysia it was found that there were no parasites present in long-tailed macaques living in urban areas whereas monkeys caught in the forested areas were infected with simian malaria parasites [28].

In PPSRNP, various wild macaque troops were observed and samples were obtained from 3 different troops. A limitation of the present study is that the sample size of 95 monkeys and 45 malaria-positives (with 40 from PPSRNP) is relatively small. Nevertheless, differences in prevalence of malaria parasites were observed between the troops. The lower prevalence of each *Plasmodium* species in troop 2 compared to the other two troops could be the effect of the choice of each troop’s sleeping sites. Troop 2 was observed to sleep on the trees approximately 4 m away from the sea front. In contrast, Troop 1 and 3 were observed to sleep inside the forests, and hence were probably more vulnerable to mosquito species which tend to prefer shaded areas in the forest as breeding sites [29].

The presence of the multiple species of *Plasmodium* detected in the macaques by nested PCR assays confirms the previous observations of the complex nature of simian *Plasmodium* parasites in long-tailed macaques [17, 26, 27, 30]. The prevalence of multiple infections in wild long-tailed macaques (86.6%) was observed to be lower in this study compared to the 92.6% observed for macaques in Sarawak, Malaysian Borneo [26], but is higher than that found in studies conducted in Peninsular Malaysia (74.3%) [30] and Singapore (42.42%) [27]. Because of this complexity of mixed species infections, it is very difficult to accurately identify the *Plasmodium* species infecting long-tailed macaques through microscopic examination alone, since the early trophozoites of all the simian malaria parasites and the late trophozoites of some species of *Plasmodium* are morphologically identical, making it difficult to differentiate one species from another [2]. In a report from Taiwan, molecular analysis confirmed that the previously thought *P. knowlesi* and *P. cynomolgi* isolates where actually *P. inui* [10], underscoring the importance of using molecular detection methods for identification of the various species of *Plasmodium*.

In the present study, *P. inui* was the most prevalent *Plasmodium* species detected, similar to what was found in the studies conducted in Sarawak and Sabah in Malaysian Borneo, and in Selangor, Peninsular Malaysia, respectively [26, 30]. The species was also found to have the highest prevalence in a study of macaques in Thailand [31], but the lowest in the study of long-tailed macaques conducted in Singapore [27]. In the present study, *P. fieldi* showed a higher prevalence compared to *P. cynomolgi, P. knowlesi* and *P. coatneyi,* while it was not detected in a previous study of long-tailed macaques from Batangas, Zamboanga and Palawan in the Philippines [17, 19]. The reasons for this observation are unclear since similar PCR assays were used in previous studies [17, 26, 27, 30] and this further highlights that there are major differences in the prevalence of each of the simian *Plasmodium* species in macaques from different geographical locations. *Plasmodium cynomolgi* also showed a relatively high prevalence in PPSRNP and this species has been reported to naturally infect humans [8, 9]. *Plasmodium cynomolgi, P. inui* and *P. coatneyi* were recorded in macaques in Palawan through microscopy several decades ago [19] and molecular identification done in the current study confirmed that these parasites are indeed currently occurring in long-tailed macaques in the island of Palawan. Moreover, for the first time, the current study records the existence of *P. fieldi* in the country, indicating that long-tailed macaques on the island of Palawan, Philippines are natural hosts for *P. cynomolgi, P. inui, P. coatneyi, P. fieldi* and *P. knowlesi*.

The presence of *P. knowlesi* and the other potentially zoonotic simian *Plasmodium* parasites such as *P. cynomolgi* [8] and *P. inui* species [32, 33] are potential threats to the local people in Palawan. The first report of human knowlesi malaria cases in Palawan was made by Luchavez et al. in 2008 [20] of 5 human cases occurring in 2006 and 2007, including one case in San Miguel, which is only 30 km from PPSNRP. Subsequently two travelers from Taiwan and USA to Palawan island were found to be infected with *P. knowlesi* in 2009 [21]. There have been no other human knowlesi malaria cases reported in the local population of Palawan Island since 2008 despite 54,314 malaria cases being detected by microscopy in the province from 2009 to 2017; 76% *P. falciparum*, 16.5% *P. vivax*, 1.4% *P. malariae*, 3.4% mixed species and 2.6% with no data of *Plasmodium* species available (unpublished data from Kilusan Ligtas Malaria [Movement Against Malaria], Provincial Government of Palawan). The lack of reports of *P. knowlesi* cases during this period among the local population is most probably due to the use of microscopy rather than molecular detection methods for routine diagnosis of malaria in Palawan Island. Misdiagnosis of *P. knowlesi* as *P. falciparum* or *P. malariae* by microscopy could have occurred since the early trophozoites of *P. knowlesi* resemble those of *P. falciparum* while the mature blood stages and gametocytes of *P. knowlesi* are similar to those of *P. malariae* [6]. In Malaysia, where the local population is infected with *P. knowlesi*, *P. falciparum* and *P. vivax*, infections with *P. knowlesi* have not only been misdiagnosed as *P. malariae* or *P. falciparum* but also as *P. vivax* by microscopy [4, 34, 35]. Although there have been no local human knowlesi malaria cases reported in Palawan island since Luchavez and co-workers reported the five cases in 2008 [20] and the travelers from Taiwan and USA who acquired knowlesi malaria the following year, there was a recent case report of a Japanese man who got infected with *P. knowlesi* during his 3 month stay at a forest resort Palawan in 2018 [22]. He was diagnosed 5 days upon his return to Japan, where a blood film revealed parasites resembling *P. malariae* or *P. knowlesi*, and a diagnosis of *P. knowlesi* was made following analysis by nested PCR assays. This strongly suggests that local cases of knowlesi malaria in Palawan have been occurring since 2008 but have been misdiagnosed by microscopy as *P. falciparum* or *P. malariae*. Given that long-tailed macaques in Palawan island are infected with *P. knowlesi* and *P. cynomolgi*, there is a need to use molecular detection methods to undertake large scale epidemiological studies in Palawan island to determine the true prevalence of zoonotic malaria among the local population. Entomological studies also need to be conducted to identify the mosquito vectors responsible for transmission of simian malaria.

Deforestation and agricultural expansion are identified as the key drivers of *P. knowlesi* infection in humans [36]. This happens when natural habitats of the reservoir hosts are reduced or the feeding behavior of the vectors of the parasites are altered which will eventually result in high transmission of the parasite from one host to another. Long-tailed macaques are said to increase density as response to deforestation, which increases the possibility of contact from one individual to another [36] and will encroach human in settlements [28]. Although PPSRNP is a protected area and deforestation is prohibited, there is a visible increase of human-macaque interaction in the area due to tourism and other activities by the locals, hence posing threats of zoonotic transmission of both *P. knowlesi* and *P. cynomolgi* to the local population.

## Conclusion

The presence of *P. knowlesi*, *P. cynomolgi, P. inui* and *P. coatneyi* in long-tailed macaques in Palawan island was confirmed using molecular detection assays. The study also reports the first detection of *P. fieldi* in macaques in the Philippines. The prevalence of *Plasmodium* spp. varied among the sites of collection and even among troops of wild macaques from one site. The presence in wild macaques of these simian *Plasmodium* parasites, especially *P. knowlesi* and *P. cynomolgi*, presents risks to the local people in Palawan island.

## Abbreviations

BMB: Biodiversity Management Bureau
IACUC: Institutional Animal Care and Use Committee
NWRRC: National Wildlife Rescue and Rehabilitation Center
PCSD: Palawan Council for Sustainable Development
PPSRNP: Puerto Princesa Subterranean River National Park
PWRCC: Palawan Wildlife Rescue and Conservation Center
PCR: polymerase chain reaction
